# Beyond the Screen, Back to Basics: Reviving In-Person Handover in Inpatient Psychiatry

**DOI:** 10.1192/bjo.2026.11403

**Published:** 2026-06-30

**Authors:** Anjali Jha, Alice Hyde, Alexandra Marsden, Joy Arthur, Catherine Ollerhead

**Affiliations:** Avon and Wiltshire Mental Health Partnership NHS Trust, Bristol, United Kingdom

## Abstract

**Aims:**

The need for a clear, consistent handover process was identified amongst resident doctors covering Avon and Wiltshire Mental Health Partnership (AWP) Bristol inpatient sites, including at Callington Road Hospital (CRH).

Previously, the overnight doctor handed over to the daytime ward doctors using a variety of methods. This included email, face-to-face, or by ward phone. Face-to-face handovers were infrequent as it was impractical to visit 7 wards individually to hand over. Daytime doctors were difficult to reach by phone, due to concurrent morning MDT handovers.

Email was a widely used handover method. However, there was risk of delay in receiving and acting on information, with patient safety implications. Our aim was to make the inpatient handover system safer and more fit-for-purpose.

**Methods:**

Our scoping questionnaire (December 2024–March 2025) found that most responders favoured face-to-face when giving (10 of 16) and receiving (11 of 16) handover. Only 25% agreed the existing system was fit-for-purpose and 19% agreed it was safe.

Based on this information, a weekday in-person handover was introduced at CRH from 9.00–9.15am.

We received backing from the Head of Bristol Inpatients, the Bristol, North Somerset and South Gloucestershire Medical Inpatient Lead, and CRH consultants to initiate a 2-month pilot.

The pilot was advertised amongst resident doctors and launched in May 2025. Feedback was gathered via online questionnaire and handover attendance monitored using a logbook.

**Results:**

82% attendance was achieved at the in-person handover during the 2-monthpilot (May–July 2025).

The 10 questionnaire responses during the pilot showed 80% agreed the new system was fit-for-purpose and 100% agreed it was safe.

Positive feedback included that there was a reduced chance of miscommunication, clarification was easier, and tasks were less likely to be missed. Night doctors found the new handover quicker, less stressful, and felt tasks were more likely to be actioned. Finally, resident doctors found the in-person handover more sociable. Suggestions for improvementincluded encouraging increased attendance, making locums aware of the new handover, and including the other 2 inpatient sites covered overnight in the handover process.

**Conclusion:**

Overall, the in-person handover pilot was successfully implemented, with overall good attendance and positive feedback regarding its safety, efficacy, and social aspect. It has been incorporated into the locum induction and induction for resident doctors working at CRH for future rotations. We also plan to extend this project to the other 2 Bristol inpatient sites to create a streamlined handover system to positively impact patient safety.

